# Dysfunction and Pathological Origins of Lymphatic Endothelial Cells in Atherosclerosis Revealed by Single-Cell Transcriptomics

**DOI:** 10.3390/genes16121398

**Published:** 2025-11-21

**Authors:** Qinhang Shen, Guangchao Gu, Dan Yang, Yuehong Zheng

**Affiliations:** 1 Department of Vascular Surgery, Peking Union Medical College Hospital, Chinese Academy of Medical Sciences and Peking Union Medical College, Beijing 100730, China; sqh21@mails.tsinghua.edu.cn (Q.S.); guguangchao@pumch.cn (G.G.); 2 Department of Computational Biology and Bioinformatics, Institute of Medicinal Plant Development, Chinese Academy of Medical Sciences and Peking Union Medical College, Beijing 100193, China; dyang0226@163.com

**Keywords:** lymphangiogenesis, atherosclerosis, lymphatic endothelial cells

## Abstract

**Background:** Atherosclerosis, a leading cause of cardiovascular disease, involves complex interactions between vascular and immune cells. The role of lymphatic endothelial cells (LECs) in this process remains incompletely characterized, limiting our understanding of disease mechanisms. This study aimed to delineate the phenotypic and functional dynamics of LECs during atherosclerosis progression. **Methods:** We performed single-cell RNA sequencing on aortic cells from ApoE-/- mice on a high-fat diet at baseline, 8 weeks (early disease), and 16 weeks (late disease). Bioinformatic analyses, including clustering, differential expression, trajectory inference, and cell-cell communication analysis, were applied to characterize LEC subpopulations and their transcriptional reprogramming. **Results:** Our analysis identified two LEC subpopulations that exhibited a biphasic numerical response: expansion at the early stage followed by a decline by the late stage. Early-disease LECs displayed altered immunomodulatory capacity, with features of reduced T cell tolerance and enhanced activation via IL-7/IL-7R signaling, coupled with a downregulation of key lipid-handling genes (*Ldlr*, *Abca1*). Trajectory analysis suggested multiple cellular origins, including a conventional but delayed differentiation path from vascular endothelial cells and an atherosclerosis-specific transdifferentiation path from fibroblasts observed only in early disease. **Conclusions:** Our findings indicate that LECs undergo substantial phenotypic and functional alterations during atherosclerosis. The maladaptive differentiation and acquired dysfunction in lipid transport and immune regulation may contribute to disease progression. This study provides a foundational transcriptional atlas for understanding lymphatic involvement in vascular disease and highlights potential contexts for therapeutic modulation.

## 1. Introduction

Atherosclerosis (AS), a chronic inflammatory disease of the arterial wall, is primarily driven by the accumulation of apolipoprotein B-containing lipoproteins in the subendothelial space [1,2]. This lipid deposition triggers a complex pathological cascade involving endothelial dysfunction, monocyte recruitment and differentiation into macrophages, and the formation of foam cells through unchecked uptake of modified lipids [3,4]. The ensuing inflammatory response, characterized by the involvement of various immune cells and cytokines, ultimately leads to plaque progression, remodeling, and potential clinical complications such as ischemia and thrombosis.

Beyond the arterial wall itself, recent evidence has highlighted the role of the lymphatic vasculature as a critical player in atherosclerosis [1,5]. Previous studies have demonstrated that lymphatic vessels undergo lymphangiogenesis within the atherosclerotic aorta and its draining lymph nodes [6]. At the same time, this observation suggests a potential compensatory mechanism; the functional consequences of this lymphatic remodeling in the context of disease progression remain incompletely understood. Theoretically, an efficient lymphatic circulation could confer atheroprotection through two key mechanisms: first, by facilitating the reverse cholesterol transport (RCT) of excess cholesterol [7] and inflammatory mediators from the plaque to the systemic circulation; and second, by modulating the immune response through the trafficking of antigen-presenting cells and the clearance of chemokines, thereby regulating local inflammation [8].

Despite these plausible hypotheses, a significant knowledge gap persists. The specific mechanisms by which lymphatic function—particularly its capacity for lipid transport and immunomodulation—influences AS are poorly defined. A major obstacle has been the limited understanding of the lymphatic endothelial cells (LECs) that form the lining of the capillary lymphatics, the primary site of interaction with the plaque microenvironment. The functional state, heterogeneity, and specific contributions of LECs to AS pathogenesis have been largely unexplored.

To address this gap, our study provides a high-resolution transcriptional landscape of cells within atherosclerotic aortic tissues in mice by analyzing single-cell RNA sequencing (scRNA-seq) results, with a dedicated focus on the LEC population. We aim to precisely characterize the association of LEC phenotypic changes throughout AS progression. Furthermore, we will investigate the dynamic functional adaptations of LECs, specifically evaluating their critical roles in cholesterol transport and the regulation of immune cell activity. This approach will yield novel insights into the multifaceted functions of LECs and elucidate their potential as a therapeutic target for modulating atherosclerosis.

## 2. Materials and Methods

**Figure 1:**
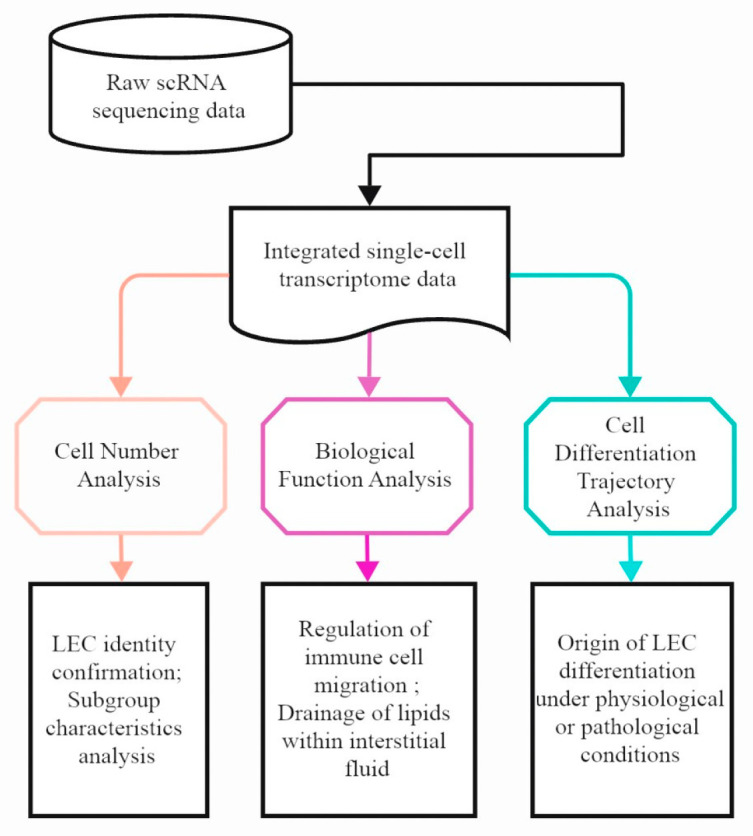
The workflow diagram shows the process of obtaining and processing the single-cell RNA sequencing data of a mouse atherosclerotic plaque. Sham: not included in high-fat-diet (HFD) group; AS1-8 W: atherosclerotic mice fed an HFD for 8 weeks; AS2-16 W: atherosclerotic mice fed an HFD for 16 weeks.

The research flow of this study is summarized in Figure 1.

### 2.1. Single-Cell RNA-seq Data Acquisition and Processing

Publicly available single-cell RNA sequencing data were obtained from the Gene Expression Omnibus (GEO) under accession number GSE131780. Our analysis specifically utilized the mouse (GSE131776) and human (GSE131778) sub-datasets from this repository. For the murine analysis, we focused exclusively on wild-type samples to avoid potential confounding effects from genetic manipulations. A total of 11 GEO samples (GSMs) were included: GSM3819841, GSM3819842, GSM3819853, GSM381944, GSM389845, GSM381984, GSM3819947, GSM381988, GSM381989, GSM381985, GSM381950, and GSM3819851. These samples represented two major cell lineages—smooth muscle cells (SMCs) and non-SMCs—across three experimental time points: baseline (sham) and after 8-week (8 w) and 16-week (16 w) high-fat-diet interventions.

All mouse samples were derived from ApoE−/− mice on a C56BL/6 background. Detailed protocols for tissue processing and cell dissociation have been previously described [9]. Briefly, immediately after sacrifice, mice were perfused with PBS. The aortic root and ascending aorta, up to the level of the brachiocephalic artery, were excised for single-cell preparation.

The gene expression matrices from all 11 samples were simultaneously read using the ‘Read10X’ function from the Seurat package (v4.4.1), which concatenates the count matrices from multiple directories. This created a unified count matrix containing all cells from all selected samples. We then created a single Seurat object using the ‘CreateSeuratObject’ function with standard quality control thresholds (min. cells = 3, min. features = 200).

Sample metadata, including original sample identity and experimental group assignment, were added to the Seurat object for downstream batch correction and differential expression analysis. Cell populations were annotated based on differential gene expression analysis, with the identity of lymphatic endothelial cells (LECs) confirmed. Subsequent analyses focused on three aspects: changes in LEC subpopulation quantity, alterations in biological functions, and cell differentiation trajectories. In the context of atherosclerosis (AS), LEC biological variations were systematically examined. Biological functional analysis emphasized the role of LECs in regulating immune cells (particularly T cells) and lipid transport. Trajectory analysis investigated the potential cellular origins of LECs under both physiological and pathological conditions.

Human coronary artery samples were dissected from explanted hearts of transplant recipients. The proximal to mid-right coronary artery (RCA) was identified, excised, and cleaned of peri-arterial fat. After excluding stented areas, atherosclerotic lesions—ranging from mild, non-calcified plaques to advanced calcified lesions—were identified. These areas were cut into approximately 50 mg sections and digested in an enzymatic cocktail containing Liberase TM and elastase. For each patient, approximately 3–6 atherosclerotic sections were processed for single-cell analysis [9].

Human scRNA-seq data (GSE131778) were analyzed independently using a consistent bioinformatic pipeline, as described for the murine data. This analysis focused on characterizing the cellular composition of atherosclerotic RCAs and specifically identifying the distribution of the LEC subpopulation in human AS.

### 2.2. Data Integration Analysis

The integrated dataset was processed and analyzed using the Seurat package (v4.4.1) in R [10]. Initial quality control involved filtering out cells expressing fewer than 500 genes and genes detected in fewer than five cells. Potential outliers were identified by assessing the number of genes, unique molecular identifiers (UMIs), and the percentage of mitochondrial genes per cell. To mitigate the effect of multiplets, cells containing more than 3500 genes were excluded. Cells with over 7.5% mitochondrial gene content were also removed to ensure analysis of high-quality cells.

Gene expression values were normalized using a library-size normalization approach, where the raw counts in each cell were scaled by the total number of reads in that cell. The normalized values were then multiplied by 10,000 and log-transformed. Highly variable genes were selected for downstream analysis. To correct for batch effects across samples, Harmony integration(harmony_1.2.0) was applied to the scaled data prior to dimensionality reduction [11]. Principal component analysis (PCA) was performed, followed by graph-based clustering in the harmonized PCA space. Finally, a two-dimensional visualization of the clusters was generated using t-distributed stochastic neighbor embedding (t-SNE).

### 2.3. Differentially Expressed Genes

The features of differentially expressed genes in the cell clusters (or subclusters) in all scRNA-seq analyses were identified with the Seurat function FindAllMarkers, with a minimum log2 (fold change) threshold of 0.25, a minimum of 0.25 fractions in cells, and an adjusted *p* value of <0.05. The *p* values were calculated based on a Wilcoxon rank-sum test and adjusted with the Benjamini–Hochberg procedure.

### 2.4. LEC Sub-Clustering

To investigate the heterogeneity within the lymphatic endothelial cell (LEC) population, we isolated all cells annotated as LECs from the integrated dataset based on their expression of canonical markers (*Lyve1*, *Pdpn*, *Prox1*, *Flt4*). This subset of cells was extracted and subjected to a separate, unsupervised clustering analysis. Briefly, the Seurat object containing only LECs was re-processed using the NormalizeData() function, followed by the identification of the top 2000 variable features using the FindVariableFeatures() function (selection.method = “vst”). Scaling was performed using ScaleData() on all genes. Linear dimensional reduction was carried out via Principal Component Analysis (PCA) with 30 principal components. To mitigate potential batch effects, we applied harmony batch correction using the RunHarmony() function, grouping cells by sample of origin (“orig.ident”). Non-linear dimensional reduction was performed using UMAP, and cells were clustered using a shared nearest neighbor (SNN) modularity optimization-based algorithm on the Harmony-corrected dimensions 1 to 20, with a clustering resolution parameter of 0.2.

### 2.5. Gene Signature Score

To analyze changes in lipoprotein and cholesterol absorption, as well as in transport-related genes, in the lymphatic endothelial cells of atherosclerotic mice, gene signature scores for lipid metabolism and transport processes were calculated using the AddModuleScore function in Seurat. Two distinct gene signatures were defined:

Lipid metabolism signature: Comprising 14 genes involved in lipid processing, storage, and regulation: *‘Lpl’*, *‘Lipe’*, *‘Pnpla2’*, *‘Pparg’*, *‘Srebf1’*, *‘Scd1’*, *‘Fasn’*, *‘Acaca’*, *‘Cpt1a’*, ‘*Abca1’, ‘Abcg1’*, *‘Soat1’*, *‘Ldlr’*, and *‘Vldlr’*.

Lipid transport signature: Comprising 12 genes encoding apolipoproteins, lipid transporters, and lipid-binding proteins: *‘Apob’*, ‘*Apoe’*, *‘Apoc1*’, ‘*Apoc2’*, ‘*Apoc3’*, *‘Mttp’*, *‘Cd36’*, ‘*Scarb1’*, *‘Fabp4’*, *‘Fabp5’, ‘Npc1’*, and *‘Npc2’*.

The combined gene set (26 unique genes) was filtered to include only genes expressed in our LEC dataset. Signature scores were calculated separately for each gene set to quantify the relative activity of lipid metabolic and transport pathways across LEC subpopulations.

### 2.6. Pathway Enrichment Analysis

Functional annotation of differentially expressed genes was carried out through Gene Ontology (GO) enrichment analysis [12]. Specifically, genes that were significantly upregulated in specific cell subpopulations or under different treatment conditions were selected as input. These gene lists were interrogated against the Biological Process (BP) domain of the GO database using clusterProfiler (v4.12.6) [12]. Significantly over-represented terms were identified based on a hypergeometric test, with the resulting *p* values adjusted for multiple comparisons using the Benjamini–Hochberg method. Terms with an adjusted *p* value (FDR) < 0.05 were considered statistically significant, providing a systematic and unbiased interpretation of the biological functions and processes most pertinent to the observed transcriptional changes.

### 2.7. Cell-Cell Communication Analysis

A comprehensive analysis of intercellular signaling networks was conducted to infer potential ligand–receptor interactions between different cell types. This analysis was primarily performed using CellChat (v1.6.1) [13], which leverages a curated database of ligand–receptor interactions and employs a robust statistical framework to model the probability of communication between cell groups based on scRNA-seq expression data. We employed the mouse-specific ligand–receptor database (CellChatDB.mouse). Communication probabilities were computed using default parameters, with the trim mean method for gene expression aggregation. Significant interactions were filtered using a minimum cell threshold of 10 cells per cell group. Only interactions with *p* values < 0.05 (calculated by permutation test) were considered statistically significant.

To gain deeper mechanistic insight into the downstream effects of specific signaling events on recipient cells, the analysis was supplemented with Commonpath [14]. This approach predicts which incoming signals are most likely to regulate the observed gene expression changes in a target cell population, thereby linking specific ligands to key signaling pathways. Key outputs included the identification of globally over- or under-active signaling pathways and the visualization of communication networks.

### 2.8. Cell Trajectory Analysis

To reconstruct cellular differentiation trajectories and model dynamic transcriptional transitions, pseudo-temporal ordering analysis was applied using Slingshot [15]. Slingshot was first used to infer global lineage structures directly from the low-dimensional embedding (t-SNE), defining initial cluster-based lineages and simultaneously assigning each cell a pseudotime value along these lineages. Subsequently, a more detailed graph-based model of the cell state manifold was built. This allowed for the ordering of cells along inferred trajectories, the identification of branch points representing fate decisions, and the analysis of genes that are dynamically regulated as a function of pseudotime.

This computational approach was applied based on the rationale that the discrete time points sampled (baseline, 8-week, and 16-week) represent critical nodes in disease progression, and our prior analyses indicated substantial and dynamic transcriptional reprogramming of LECs across these stages. We reasoned that trajectory inference could help elucidate whether these observed discrete changes were part of a broader, continuous adaptive process. The results provided a robust and high-resolution understanding of developmental dynamics and potential cellular origins, particularly for the lymphatic endothelial cell (LEC) subpopulations.

## 3. Results

### 3.1. The Single-Cell RNA Sequencing Results Revealed Endothelial Cell Heterogeneity in Mouse Aortic Atherosclerotic Plaques

**Figure 2:**
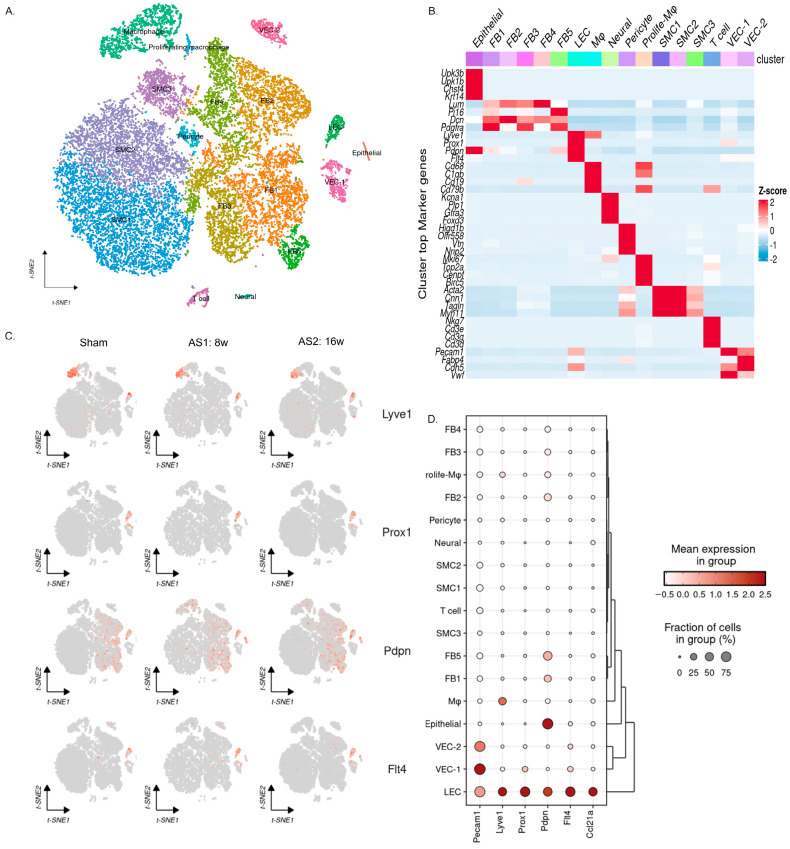
LEC identity confirmation. (**A**) t-SNE plot showing the clustering of all cells after integration, with a total of 17 cell subgroups. (**B**) Heatmap displaying the expression of marker genes in each subgroup. (**C**) Feature plot displaying the expression distribution of LEC marker genes across different groups (Sham, AS-8 w, AS-16 w). (**D**) Dot plot showing the distribution of LEC marker gene expression among the 17 cell subgroups.

After integrating the single-cell RNA sequencing results obtained from the atherosclerotic plaques of mice across different treatment groups (sham, *n* = 3; 8-week high-fat diet, *n* = 3; and 16-week high-fat diet, *n* = 3) and performing quality control, a total of 21,479 cells were retained. We then analyzed the cell-type composition of the plaque tissue. Through machine-learning-based clustering analysis, we identified 17 cell subpopulations (Figure 2A). By examining the expression of cell-type-specific marker genes (Figure 2B, macrophages: *Cd68*, *C1qb*, *Lyz2*, *Cd14*; smooth muscle cells (SMCs): *Acta2*, *Cnn1*, *Tagln*, *Myh11*; fibroblasts (FBs): *Lum*, *Pi16*, *Dcn*, *Pdgfra*; endothelial cells (ECs): *Pecam1*, *Fabp4*, *Cdh5*, *Cldn5*; T cells: *Cd3g*, *Cd3e*, *Cd3d*; neural cells: *Kcna1*, *Plp1*; proliferating cells: *Mki67*, *Top2a*, *Cenpf*, *Birc5*), we ultimately identified nine cell types present in the tissue, including smooth muscle cells, fibroblasts, endothelial cells, macrophages, T lymphocytes, neural cells, epithelial cells, proliferating macrophages, and pericytes (Figure 2B). The cell group annotated as “Mφ” here expresses a powerful set of macrophage markers, but at the same time, this group of cells shows detectable expression of B cell genes such as *Cd19* and *Cd79*. This may indicate the presence of cell double-stranded bodies (such as macrophage B cells) within the cluster, which is a known technical artifact in scRNA-seq that is difficult to completely filter out.

To further determine the proportion of lymphatic endothelial cells (LECs) in atherosclerotic plaque tissue, we examined the distribution of vascular-endothelial-specific genes and lymphatic-endothelial-specific genes across these 17 cell subpopulations (Figure 2C). The expression of CD31(also called Platelet endothelial cell adhesion molecule, shortened as PECAM-1) was notably significant in three subpopulations. One of these subpopulations also shows high expression of lymphatic endothelial-specific genes (*Lyve1, Prox1, Vegfr-3* (also known as *Flt4*), Pdpn). Therefore, we can preliminarily conclude that this cell cluster includes LECs, which are traditionally defined as *CD31^+^Lyve1^+^* cells (Figure 2D).

### 3.2. The Expression of Lymphatic-Endothelial-Specific Genes Suggests the Presence of Multiple Cell Populations Associated with Lymphatic Endothelium

In order to define the identity of LECs in plaque tissue, we utilized four genes (*Lyve1, Prox1, Vegfr-3, Pdpn*) that are highly relevant to LECs and have been widely recognized as LEC marker genes. Interestingly, we noticed that these four lymphatic-endothelial-specific genes are not exclusively expressed in LECs. Further examination revealed that five cell populations presented variant expression levels of these four lymphatic-endothelial-specific genes, including fibroblasts (FBs), smooth muscle cells (SMCs), macrophages, vascular endothelial cells (VECs), and lymphatic endothelial cells (LECs, Figure 2C). Among these, only LECs showed high expression of all four LEC-specific genes (*Lyve1, Prox1, Vegfr-3, Pdpn*). In FBs, the LEC-specific gene with the highest proportion of expression was *Pdpn* (expression proportion = 20.26%), while *Lyve1*, *Prox1*, and *Vegfr-3* were expressed only sporadically. In SMCs, the expression proportions of these four genes were similar and relatively low. In macrophages, the LEC-specific gene with the highest proportion of expression was *Lyve1* (expression proportion = 35.00%), followed by *Pdpn* (expression proportion = 5.83%). In VECs, the gene with the highest proportion of expression was *Prox1* (expression proportion = 10.1%); the other three genes were also expressed to a certain extent. These results suggest that the expression of a single LEC-specific gene is not sufficient to identify LECs. Multiple genes need to be co-localized for accurate identification. Furthermore, the partial expression of certain LEC-specific genes in the other four cell populations (except for lymphatic endothelial cells) hints at functional and differentiation similarities or association with LECs.

### 3.3. Dynamic Changes in LEC Abundance Suggest Lymphangiogenesis in Early-Stage Atherosclerosis

**Figure 3:**
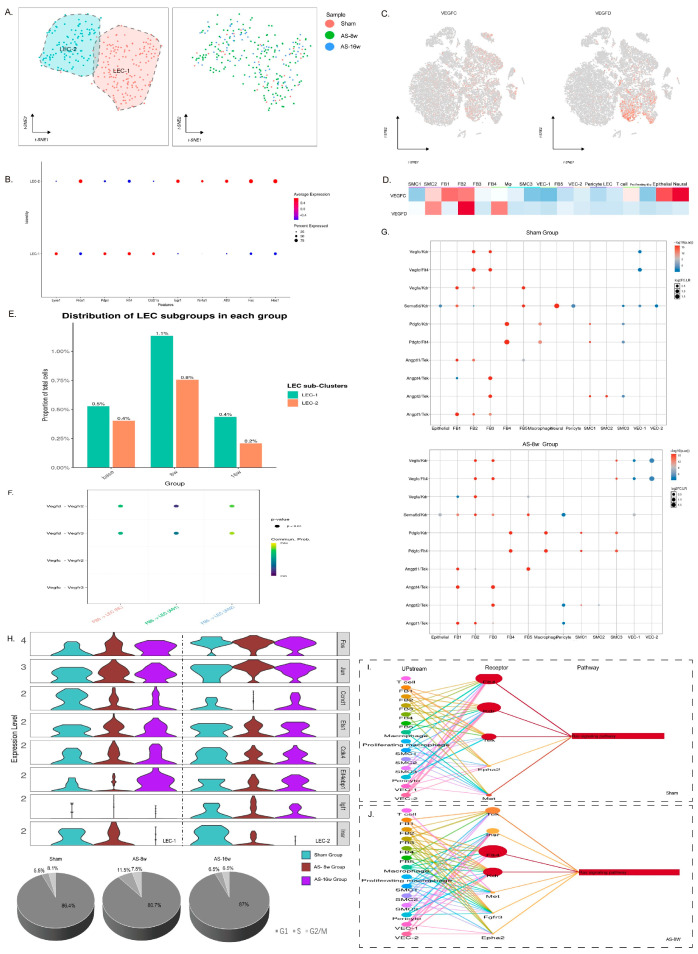
Changes in the number of lymphatic endothelial cells and analysis of proliferation-related signals. (**A**) (**Left**): t-SNE plot showing the LEC subpopulations (LEC-1 and LEC-2); (**Right**): Intergroup variations of LEC subpopulations. (**B**) Dot plot showing the top 6 genes expressed in each LEC subpopulation. (**C**) Feature plot showing the expression distribution of VEGF-C and VEGF-D in all clusters (samples were integrated together: Sham+AS8 w+AS16 w). (**D**) Heatmap showing the main VEGF-C and VEGF-D secreting subpopulations. (**E**) Analysis of LEC proportion between groups (The percentage represents the proportion of each LEC subgroup in the total number of cells in each group); the proportion of LECs increases in the early stages and decreases in the late stages of the disease. (**F**) Analysis of the interaction intensity of VEGF-C/D between cell subpopulations across groups (NL = Sham, AS1 = AS1:8 w HFD, AS2 = AS2:16 w HFD). (**G**) Top: RAS signaling activation receptor–ligand pair analysis in LEC subpopulations in the Sham group (the vertical axis represents receptors that convey activated signals, and the horizontal axis represents upstream cell subpopulations expressing the corresponding ligands); Bottom: RAS signaling activation receptor–ligand pair analysis in LEC subpopulations of the AS-8 w group. (**H**) LEC proliferative activity analysis: Top: Violin plot showing the intergroup differences in the expression of cell-cycle-related genes in each LEC subpopulation; Bottom: LEC cell cycle scores within each group. (**I**) Analysis of LEC proliferation-related activation pathways and upstream cell subpopulations in the Sham group (including the RAS signaling pathway, PI3K/AKT signaling pathway [16], and 5 other pathways; only the RAS signaling pathway was activated). (**J**) Analysis of LEC proliferation-related activation pathways and upstream cell subpopulations in the AS-8 w group (including the RAS signaling pathway, PI3K/AKT signaling pathway, and 5 other pathways; only the RAS signaling pathway was activated).

Sub-clustering of the lymphatic endothelial cell (LEC) population revealed two distinct subsets, suggesting a pre-existing division. The LEC-1 subset was characterized by high expression levels of *Lyve1* and *Ccl21*, whereas the LEC-2 subset had elevated expression of *Prox1* and *Bmp4* (Figure 3A,B). Despite their transcriptional differences, the two subsets exhibited a synchronized quantitative response to disease progression. A notable doubling in each LEC subcluster was observed during the early stage (8 weeks), which was followed by a significant reduction in the late stage (16 weeks) (Figure 3E). Cell-cycle-phase scoring corroborated this dynamic; while most LECs resided in the G1 phase under normal conditions, the proportion of LECs in the S phase increased substantially in early atherosclerosis (AS-8 w), coinciding with the upregulation of key cell-cycle-related genes (Figure 3H and Figure S1). This indicates enhanced proliferative capacity and suggests active lymphangiogenesis in the aortic wall during the early stages of the disease. However, this proliferative state was not sustained. By the late stage (AS-16 w), the proportion of S-phase LECs sharply decreased, accompanied by a decline in cell cycle gene expression (Figure 3H). The eventual reduction in LEC abundance corresponds to a decrease in lymphatic vessel density in advanced plaques, suggesting a collapse of the lymphatic network.

### 3.4. Activation of the RAS Signaling Pathway Is a Key Driver of LEC Proliferation

The acquisition of a proliferative state in LECs is governed by extrinsic signaling cues. VEGF-C has long been recognized as the main signal for inducing lymphangiogenesis. It functions by binding to VEGFR-3 on LECs. VEGF-D has also been reported to induce lymphatic endothelial proliferation by binding to VEGFR-3. Although VEGF-C and VEGF-D, secreted predominantly by fibroblasts and smooth muscle cells, respectively, are well-established ligands for the pro-lymphangiogenic receptor VEGFR-3 (FLT4) (Figure 3C,D), intercellular communication analysis revealed no significant increase in the incoming VEGF-C/D signaling strength to LECs during the early disease stage compared to basal conditions (Figure 3F). This prompted an investigation into alternative downstream pathways. Analysis of proliferation-related signaling pathways showed that the RAS pathway was specifically activated in LECs from normal tissues and early atherosclerotic plaques but not in LECs from late-stage disease (Figure 3I,J). Ligand–receptor pair analysis identified VEGFR-3 as the primary receptor associated with this RAS activation, with contributions from VEGFR-2 (also known as Kdr) and TEK (Figure 3G). The dissociation between the absence of increased VEGF-C/D signal input and the clear activation of the RAS pathway in early-disease LECs suggests a model where the proliferative response may be mediated through heightened sensitivity or alternative activation of VEGFR-3, rather than increased ligand availability. Furthermore, the concomitant activity changes of other receptors on LECs, such as the insulin receptor (Insr) and fibroblast growth factor receptor (Fgfr) (Figure 3H,J), indicate that non-canonical signals may also contribute to early-disease proliferative signaling in LECs, potentially converging on RAS pathway activation.

### 3.5. LEC-Mediated T Cell Migration and Activation in Early Disease Progression

**Figure 4:**
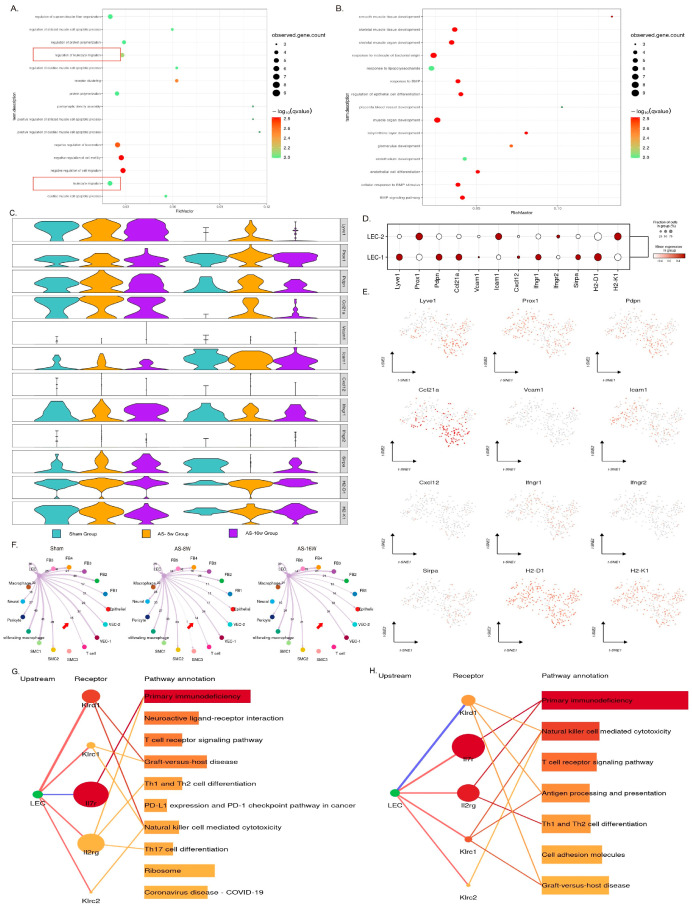
Functional analysis of LEC-mediated T cell migration and activation. (**A**) Gene Ontology (GO) enrichment analysis results (Biological Process, BP) of differentially expressed genes (DEGs) in the LEC-1 subpopulation. (**B**) GO enrichment analysis results (BP) of DEGs in the LEC-2 subpopulation. (**C**,**D**) Analysis of intergroup differences in the expression of immune cell adhesion/migration-related genes and the chemokine Ccl21 in LECs; in (**C**), left three columns: LEC-1; right three columns: LEC-2. (**E**) Feature plot displaying the expression of immune cell adhesion/migration-related genes and the chemokine Ccl21 in LECs. (**F**) Cell–cell communication analysis. Red arrows highlight interactions between LECs (upstream) and T cells (downstream). (**G**,**H**) Receptor–ligand pair analysis, with LECs as the upstream source (ligands) and T cells as the downstream target (receptors). The top ten signaling pathways ranked by interaction strength are shown (or all available if fewer than ten); (**G**) compares the Sham group and the AS1:8 w group, (**H**) compares the AS1:8 w group and the AS2:16 w group. Red indicates higher interaction strength in the former group of the comparison, while blue indicates higher interaction strength in the latter group of the comparison.

As the main cellular component of the terminal capillary lymphatic vessels, LECs directly influence the functionality of the aortic tissue’s lymphatic circulation. Previous work has identified two LEC subpopulations exhibiting gene expression differences. Notably, sub-clustering of LECs revealed two distinct subsets: LEC-1, enriched in genes related to immune chemotaxis (e.g., *Ccl21a*), and LEC-2, which expressed higher levels of developmental genes (e.g., *Bmp4*) and lipid receptors (Figure 3B). This inherent heterogeneity suggests a pre-existing division of labor: the LEC-1 subpopulation seems to be the primary executor of immune cell chemotaxis and migration in lymphatic circulation (Figure 4A,B). Interestingly, when comparing the expression of genes related to leukocyte migration and chemotaxis between different disease groups (Figure 4C–E), the change in expression levels mirrored the proliferative status of the subpopulations: there was an increase in expression during the early stage (8 w) and a decrease during the late stage (16 w). This finding suggests that in the progression of aortic atherosclerosis, the regulation of immune cells by LECs correlates with changes in their proliferative state.

Through intercellular communication analysis (Cellchat), we compared the changes in LEC and T cell interactions across different stages of the disease. In the comparison between the normal and early-stage groups (8 w), the overall communication strength from LECs to T cells did not change significantly (Figure 4F). However, from the perspective of receptor–ligand pairs, there was a notable shift in the communication pattern between LECs and T cells (Figure S3). In the normal state, LECs regulate T cell function via Galectin-9 (a lectin) binding to T cell surface molecules such as Cd45 and Cd44, limiting T cell immune responses and inducing peripheral immune tolerance. In the early stages of the disease, this limiting function of LECs on T cells gradually diminishes. Conversely, LECs mediate T cell migration through a series of surface adhesion molecules (Icam1, Icam2) and the secretion of extracellular matrix components (Fn1, Col4a2). It is worth noting that the receptor–ligand activation analysis results revealed there was greater IL-7–IL-7R interaction between LECs and T cells in the early stage compared to the basal and late-stage disease states (Figure 4G,H). This suggests that in the early stages of AS, LECs have a greater ability to activate T cells and promote immune responses by secreting IL-7.

However, by the late stage (16 w), the communication strength between LECs and T cells had sharply decreased (Figure 4F), and LECs not only lost their proliferative activity but also their ability to regulate T cell migration and activation. Compared to the early stages, the number of lymphocyte cells in aortic tissue is significantly decreased in the late stage, indicating that the lesion has transitioned from acute to chronic inflammation. This result also suggests that the regulatory function of LECs with respect to T cell migration and activation primarily occurs during the early acute inflammation phase of atherosclerosis.

Furthermore, we observed that LECs might be involved in the secretion of CCL2, CCL3, CCL4, and CXCL12. Among all cell populations, CCL2, CCL3, and CCL4 are mainly secreted by macrophages, while CXCL12 is predominantly secreted by fibroblasts (Figure S4E). Intergroup comparisons revealed that throughout the progression of the disease, the levels of a series of inflammatory chemokines secreted by proliferating macrophages were elevated, indicating that these proliferating macrophages and fibroblasts recruit monocytes and T cells to inflammatory sites via chemokine secretion. Similarly, cell–cell communication analysis demonstrated that the interactions between LECs and macrophages/fibroblasts were altered during disease progression, with LECs modulating the chemokines secreted by these upstream cells (macrophages and fibroblasts). Specifically, LECs express Ackr2 and Ackr3, which can bind to chemokines, such as CCL2, CCL3, CCL4, and CXCL12, secreted by upstream cells. Among these chemokines, LECs exhibit a stronger regulatory effect on the CXCL12 secreted by fibroblasts. Compared to the control group, LECs showed increased regulation of the CCL2, CCL3, and CCL4 secreted by macrophages in both the early and late disease stages (Figure S4F,G). These results suggest that, in addition to their direct regulatory effects on T cells, lymphatic endothelial cells may also influence the migration of immune cells to plaques by modulating the secretion of chemokines at inflammatory sites.

### 3.6. The Lipo-Transfer Function of LECs Is Disrupted in Atherosclerotic Disease

During the formation of atherosclerotic plaques, the accumulation of lipids beneath the endothelium is one of the key factors contributing to pathological changes. These excess lipids, particularly cholesterol, are engulfed by cells in the tissue, such as macrophages and endothelial cells. Once oxidized, they form cholesterol crystals, which are phagocytosed and induce foam cell formation, exacerbating the transition from early to advanced plaque stages. As the site of lymph production, lymphatic endothelial cells (LECs) are situated along crucial lipid transport pathways [5], where they take up lipid metabolites accumulated in the tissue and transport them via the lymphatic circulation. However, when comparing the molecular profile associated with the handling of lipid products—such as lipoproteins and cholesterol—in healthy aortic tissue versus early and advanced plaques, it appears that this program in LECs is impaired.

**Figure 5:**
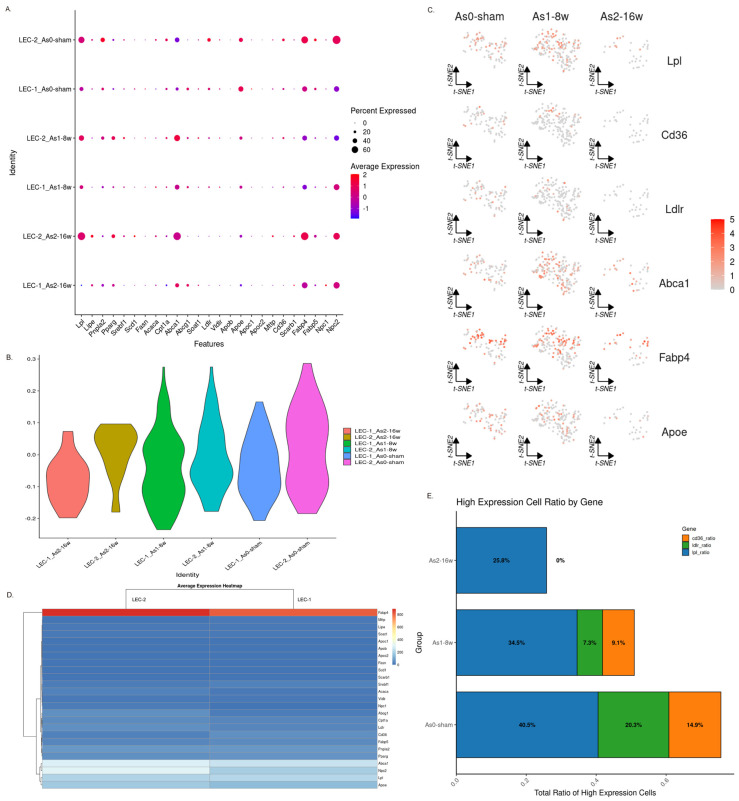
Expression analysis of LEC lipid-handling genes. (**A**) Dot plot displaying the expression of lipoprotein- and cholesterol-transport-related genes in LECs. LEC (1, 2)_(sham, AS-8 w, AS-16 w) indicates the expression changes of LEC subpopulations under different conditions. (**B**) Violin plot showing the overall expression changes of the lipid transport genes from panel A. (**C**) Feature plot (e.g., UMAP) showing the expression patterns of lipoprotein-transport-related genes. (**D**) Average expression heatmap comparing the expression of lipid metabolic and transport genes across LEC subpopulations. (**E**) Changes in the proportion of LECs with lipoprotein/cholesterol transport capability across different disease stages.

By comparing the expression levels of a series of genes involved in lipid uptake and transport (Figure 5A), including LEC scavenger receptors (*CD36*), the low-density lipoprotein (LDL) receptor, ATP-binding cassette transporter A1 (*ABCA1*), and fatty acid-binding protein 4 (*FABP4*), among others, we found that both LEC subpopulations (LEC1 and LEC2) express this molecular machinery, albeit with slight differences in expression levels between the subpopulations. Specifically, the LEC2 subpopulation exhibited higher expression levels of *CD36, Ldlr, Lpl,* and other lipid-handling genes (Figure 5B,C). When comparing the lipid-handling gene signature in LECs from early plaques and normal tissues, we observed that the score for this signature in plaque areas was significantly lower than in healthy tissue, and this reduction was even more pronounced in advanced-stage plaques (Figure 5E). These results suggest that, although LECs proliferate in the early stages of the disease, during the progression of atherosclerosis (AS), their capacity to manage lipids, as inferred from the downregulation of key genes, is compromised. The role of receptors like LDLR in LECs is likely distinct from its systemic cholesterol-clearing function in the liver and may instead be involved in local lipoprotein interaction and internalization, a process which becomes dysregulated in the plaque microenvironment.

### 3.7. The Multi-Differentiated Origin of LECs Under Pathological Conditions Cannot Compensate for the Loss of Function

**Figure 6:**
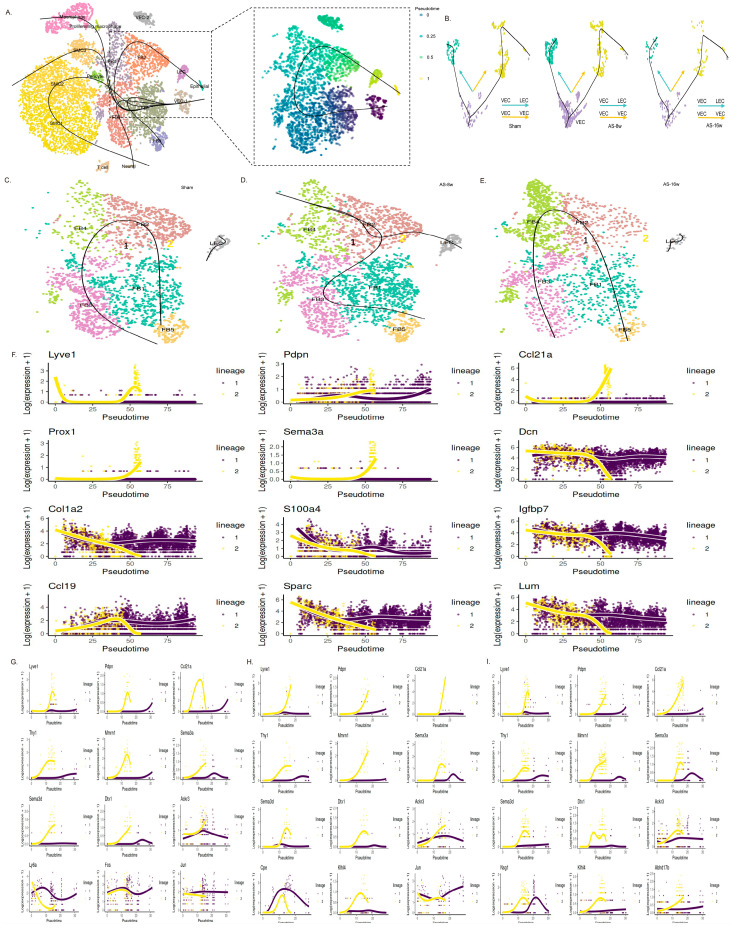
Pseudotime analysis reveals diverse differentiation trajectories of LECs under pathological conditions. (**A**) Pseudotime analysis of the integrated samples using Slingshot. The right panel shows all cells involved in the differentiation trajectory containing LECs (totaling 8 subpopulations: VEC1-2, FB1-4, LEC, and Epithelial). (**B**) Trajectory analysis results for VECs and LECs. (**C**–**E**) Trajectory analysis results for FBs and LECs. (**F**) Expression dynamics of trajectory-related genes along the pseudotime axis for the FB-to-LEC differentiation path in the AS1:8 w group. (**G**–**I**) Expression dynamics of trajectory-related genes along the pseudotime axis for the VEC-to-LEC differentiation path.

Trajectory analysis of the cells from early atherosclerotic plaques identified potential differentiation paths contributing to the expansion of the lymphatic endothelial cell (LEC) population. The inferred trajectory leading to LECs included vascular endothelial cell subpopulations (VEC-1, VEC-2), fibroblast clusters (FB1-FB4), and LECs themselves (Figure 6A). Pseudotime ordering suggested that fibroblasts were positioned closer to LECs along the differentiation continuum compared to vascular endothelial cells, indicating two potential precursor cell types (fibroblasts and vascular endothelial cells).

Further analysis of these putative paths revealed distinct contexts for each differentiation route. A differentiation trajectory from fibroblast subgroup 2 (FB) to LECs was specifically inferred in early-disease tissues (AS1:8 w), where LEC proliferation is active (Figure 6D). This FB-to-LEC path was not observed in the control (Sham) or late-disease (AS2:16 w) groups, which only showed intra-lineage differentiation (Figure 6C,E). Analysis of gene expression dynamics along the pseudotime axis confirmed this transition: expression of canonical LEC markers and functional genes (e.g., *Lyve1, Prox1, Pdpn, Ccl21*) increased towards the trajectory endpoint (pseudotime > 50), while fibroblast-specific genes involved in collagen synthesis (e.g., *Dcn, Col1a2, Lum*) were progressively downregulated and eventually silenced (Figure 6F). This transcriptional shift indicates a loss of fibroblast identity and the acquisition of a lymphatic endothelial phenotype. Notably, the expression of *Igfbp7* remained high until late pseudotime, dropping sharply as *Prox1* and *Ccl21a* expression peaked, suggesting a potential role for insulin-like growth factor signaling in this differentiation process.

In contrast to the pathology-restricted FB-to-LEC transition, a differentiation path from the VEC-1 vascular endothelial subpopulation to LECs was detected under both basal and disease conditions (Figure 6B). However, the dynamics of this VEC-to-LEC differentiation were altered in atherosclerosis. In healthy tissues, LEC-identity genes were rapidly upregulated and peaked early in the differentiation process (pseudotime > 10; Figure 6G). In disease conditions, the upregulation of these genes was delayed (pseudotime > 15), with a more pronounced delay observed in late-stage plaques (Figure 6H,I). These results suggest that while differentiation from vascular endothelial cells represents a physiological source of LECs, this process is significantly hampered during atherosclerosis, resulting in slower maturation of functionally competent LECs.

The analysis of LEC functions, including lipid phagocytosis and immune cell regulation, across different groups revealed that, compared to the healthy state, in the disease state, the lipid absorption and metabolic capacity of LECs, as well as the interaction strength with T cells, were both reduced (Supplement Figure S4H). This indicates that in AS disease, even though the differentiation sources of LECs increase and their proliferative activity is enhanced in the early stages, the newly generated LECs have partial functional impairments. Their lipid absorption and transport functions, as well as their immune regulatory capabilities, are immature and may even exacerbate the inflammatory response in atherosclerotic plaque areas. In the late stages of disease, the lack of proliferative signals (mainly VEGF-C, Figure 3F) prevents LECs from maintaining their proliferative activity and functional impairments worsen, leading to further disruption of lymphatic circulation in the affected areas.

### 3.8. Single-Cell Transcriptomic Profiling Reveals a Scarcity of Lymphatic Endothelial Cells in Human Atherosclerotic Coronary Arteries

**Figure 7:**
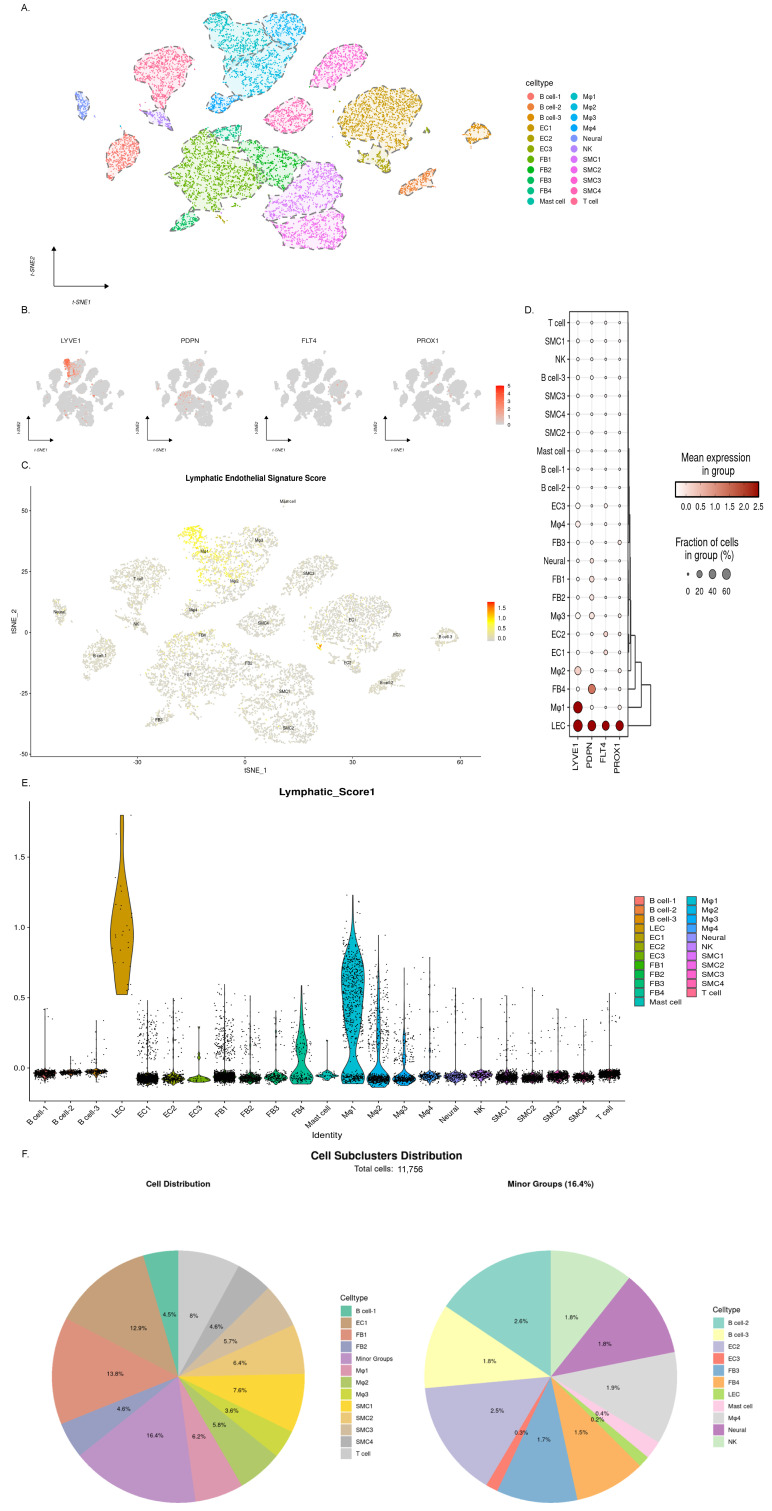
Distribution of lymphatic endothelial cells (LECs) in human atherosclerotic coronary arteries. (**A**) t-SNE projection showing all cell clusters from the proximal to mid-segment of the right coronary artery. Cell clusters are annotated based on classic marker genes: macrophages (Mφ): *‘CD68’, ‘C1QB’, ‘LYZ’, ‘CD14’*; smooth muscle cells (SMCs): *‘ACTA2’, ‘CNN1’, ‘TAGLN’, ‘MYH11’*; fibroblasts (FBs): *‘LUM’, ‘DCN’, ‘PDGFRA’*; endothelial cells (ECs): *‘PECAM1’, ‘FABP4’, ‘CDH5’, ‘CLDN5’*; T cells: *‘CD3E’, ‘CD3G’, ‘CD3D’*; B cells: *‘CD79A’, ‘CD79B’, ‘CD19’*; neural cells: *‘PLP1’, ‘MPZ’, ‘PRIMA1’, ‘FXYD3’*; mast cells: *‘TPSAB1’, ‘CPA3’, ‘MS4A2’, ‘TPSB2’*. (**B**) Feature plots showing the expression of four LEC hallmark genes (left to right: *LYVE1, PDPN, FLT4, PROX1*). *LYVE1* expression was highest and was detected in macrophages, fibroblasts, and endothelial cells. *PDPN* was highly expressed in macrophages, fibroblasts, neural cells, and endothelial cells. *FLT4* expression was largely confined to endothelial cells. *PROX1* was expressed in macrophages, fibroblasts, and endothelial cells. (**C**) LEC signature scoring. An LEC signature score was calculated using Seurat’s AddModuleScore function based on a gene set containing *LYVE1, PDPN, FLT4*, and *PROX1*. The t-SNE plot visualizes the score per cell. Cells with the highest scores were concentrated within the endothelial cluster. Macrophages and fibroblasts also contained cells with positive scores (>0) due to partial expression of *LYVE1* and/or *PDPN*. (**D**) Dot plot displaying the expression of LEC signature genes across cell subclusters. Cells from the original endothelial cluster with an LEC signature score > 0.5 were re-annotated as the LEC population. This population co-expressed all four LEC marker genes (*LYVE1, PDPN, FLT4, PROX1*) at high levels. (**E**) Violin plots showing the LEC signature score across re-annotated cell subclusters. The LEC cluster exhibited the highest score. The second-highest score was observed in Mφ1, primarily due to high *LYVE1* expression. (**F**) Proportional abundance of re-annotated cell subpopulations. Left: Proportion of all cell subclusters in the right coronary artery. ‘Minor groups’ represent the aggregate of clusters, each constituting less than 3% of the total cells. Right: Distribution analysis of cell subpopulations within the ‘minor groups’. All percentages shown are relative to the total cell count (*n* = 11,756). LECs account for approximately 0.2% of all cells.

Based on single-cell RNA sequencing data pertaining to the atherosclerotic right coronary artery from a heart transplant recipient (Figure 7), we sought to delineate the distribution of lymphatic endothelial cells (LECs) in human atherosclerosis. The expression pattern of canonical LEC marker genes in human lesions closely mirrored our observations in mice: *LYVE1* and *PDPN* were promiscuously expressed across multiple cell types, including macrophages and fibroblasts, while *PROX1* was also non-specific, and *FLT4* expression remained relatively restricted to endothelial cells (Figure 7B). This overlap underscores the challenge of relying on any single gene for definitive LEC identification in this complex microenvironment.

To overcome this limitation, we employed a computational strategy using Seurat’s AddModuleScore to calculate a composite LEC signature score based on the combined expression of LYVE1, PDPN, FLT4, and PROX1 (Figure 7C). Cells within the original endothelial cluster that had a high signature score (>0.5) were re-annotated as a distinct LEC population (Figure 7D). This redefined LEC cluster demonstrated high co-expression of all four marker genes and possessed the highest signature score, confirming its identity (Figure 7E). The cellular composition of the plaque was characterized by a substantial infiltration of immune cells, including macrophages (17.5%), T cells (8%), and B cells (7.1%), indicative of a pronounced inflammatory state. In stark contrast, the proportion of LECs was remarkably low, constituting only 0.2% of all cells (Figure 7F). This scarcity of LECs in advanced human lesions aligns with our findings of a decline in LEC numbers in late-stage murine atherosclerosis, suggesting that a deficiency in LEC abundance (and potentially function) may be a contributing factor in human AS pathogenesis.

## 4. Discussion

Our single-cell transcriptomic analysis reveals that lymphatic endothelial cells (LECs) in atherosclerosis undergo a dynamic biphasic response, shifting from an early compensatory phase to late functional decline. We identified two transcriptionally distinct LEC subsets: an immunomodulatory LEC-1 population and a lipid-handling LEC-2 population. Despite this functional heterogeneity, both subsets expanded synchronously in early disease, likely driven by RAS pathway activation, before cell numbers declined in advanced plaques. These findings align with emerging concepts of profound cellular plasticity in the atherosclerotic microenvironment and suggest that the plaque milieu actively shapes LEC ontogeny and fate [17].

A central finding is the functional impairment of LECs despite their increased cell numbers. Early in the disease, LECs transition from a tolerogenic state to actively promoting inflammation via mechanisms such as IL-7/IL-7R signaling, while concurrently losing their capacity to clear lipids. This paradoxical state—where increased LEC numbers correlate with worsened immunoregulation and lipid retention—suggests the newly generated LECs are functionally immature or dysregulated. This observation may challenge the prevailing view that lymphangiogenesis is purely beneficial in atherosclerosis [18,19,20].

Furthermore, the clinical relevance of our findings is bolstered by our analysis of human atherosclerotic coronary arteries, which not only confirmed the conserved challenge of defining LECs by single markers but also revealed an extreme scarcity of LECs (approximately 0.2%) in advanced human lesions. This consistency between mouse- and human-derived data reinforces the pathophysiological significance of LEC loss in late-stage disease and underscores the need to explore therapeutic strategies aimed at preserving or restoring lymphatic function in atherosclerosis.

The functional heterogeneity of LEC subpopulations has been previously documented. In a study profiling LEC subsets across murine lymph nodes, several distinct populations were identified, including floor LECs (fLECs), ceiling LECs (cLECs), and medullary LECs. Notably, the cLEC subset was characterized by high expression of Fabp4, Cd36, and Ackr3, bearing a transcriptional resemblance to our defined LEC-2 subpopulation [16]. Furthermore, the study provided functional evidence by demonstrating that cLECs selectively uptake and clear modified low-density lipoprotein from the lymph, a finding consistent with our conclusion linking the LEC-2 subset to lipid uptake and transport functions. As for the potential interaction between the LEC-1 subset and T cells through IL-7/IL-7R, although there are no direct experimental data to prove this, previous studies have shown that LECs are one of the main sources of IL-7 in mice [21].

Our findings that disease-associated LECs exhibit a downregulated gene signature for lipid uptake and transport have broader implications for the pathophysiology of atherosclerosis. Lymphatic vessels are believed to be involved in the reverse transport of cholesterol at the arterial wall, including pathways that enter the systemic circulation in the form of lipidated apolipoprotein A-I or HDL-C [5]. In this process, cholesterol effluxed from macrophage foam cells is expected to be taken up by surrounding LECs and transported via the lymphatic vasculature for ultimate hepatic excretion. The impaired molecular machinery we observed—including reduced expression of receptors (Scarb1) and transporters (Abca1, Abcg1)—strongly suggests a functional deficit in the lymphatic RCT pathway in advanced atherosclerosis. We propose that this LEC dysfunction creates a “traffic jam” within the plaque, where cholesterol cannot be efficiently cleared, thereby accelerating lipid accumulation and disease progression.

In the discussion of the immune function of LECs, we mainly focused on the relationship with T lymphocyte populations. The regulatory effect of LECs on T cells has been reported in previous studies. A study on hypercholesterolemia showed that lysophosphatidic acid (LPA) induces overexpression of calpain in LECs and that overactivation of calpain disrupts the stability of Tregs [22]. This inhibitory effect seems to work via the suppression of TGF-β production. Calpain degrades intracellular proteins such as SMAD2 through protein hydrolysis, thereby inhibiting TGF-β signaling. In lymphatic vessels undergoing inflammatory proliferation, the activation of the pro-inflammatory transcription factor nuclear factor kappa B (NF κ B) was also detected in LECs; this induced secretion of pro-inflammatory cytokines such as IL-18 and expression of adhesion molecules such as VCAM-1. The increases in IL-18 and VCAM-1 further modulate the differentiation and transport of T cells, exacerbating the inflammatory response [23]. Our data support a model where the quality and functionality of LECs are equally important as their quantity. The functional failure in late-stage disease is characterized by the loss of proliferation.

The expansion of LECs in early-stage disease is facilitated by a previously unrecognized pathological trans-differentiation of fibroblasts into LECs, alongside a delayed—but conserved—differentiation path from vascular endothelial cells. The differentiation trajectory from vascular endothelium to lymphatic endothelium is widely believed to be the primary origin of lymphatic endothelial progenitor cells during embryonic development [24,25], while there are few reports on lymphatic neovascularization occurring in adult individuals. Other heterogeneous LEC progenitors show differences in their organ of origin, such as the detection of erythroid progenitor cells (EMPs), which are suspected to originate from embryonic yolk sac (YS) production in the heart [26]. The differentiation trajectory of fibroblasts to lymphatic endothelial progenitor cells is detected in the current study, providing additional insight into the origin of LECs. Perhaps this change in cell identity does not really exist; however, the evidence is sufficient to suggest that the differentiation process of LECs is affected against the backdrop of atherosclerotic disease. These new LECs lack the functional characteristics of typical LECs and are more closely aligned with the secretory function of fibroblasts.

In summary, our study suggests that LEC dysfunction, driven by aberrant differentiation and functional reprogramming, is a critical contributor to the pathogenesis of atherosclerosis. The maladaptive LEC response, marked by inefficient lipid clearance and a shift toward pro-inflammatory T cell activation, likely exacerbates disease progression. This refined understanding shifts the focus from merely promoting LEC growth to therapeutically steering LEC function towards a stable, homeostatic phenotype. Future studies validating these mechanisms in human tissues and testing strategies to modulate LEC differentiation and function in vivo will be essential for translating these insights into novel therapeutic avenues for atherosclerosis.

### Limitations

While our study provides a comprehensive transcriptional landscape of LEC dynamics in murine atherosclerosis, several limitations should be acknowledged. First, the intriguing observation of fibroblast-to-LEC transdifferentiation, while supported by pseud-temporal trajectory analysis, requires formal validation through lineage-tracing models in future studies to definitively establish this cellular plasticity path in vivo. Second, the proposed mechanisms—such as the functional consequences of IL-7/IL-7R signaling on T cell activation and the mechanistic basis of lipid clearance defects—are derived from transcriptomic inference and need direct functional validation using genetic or pharmacological approaches. However, these findings align with growing evidence of remarkable cell fate plasticity in the vascular niche [27] and the emerging role of LECs as immune modulators beyond their classical drainage function [28], offering testable hypotheses for future investigation. Furthermore, the identification of a macrophage cluster with co-expression of B-cell markers suggests that technical artifacts like cell doublets, while minimized, may still be present and represent a challenge for data interpretation.

## Data Availability

The data that support the findings of this study are available from the corresponding author upon reasonable request. The scRNA-seq data from our study are available in Gene Expression Omnibus (GSE131780), “https://www.ncbi.nlm.nih.gov/geo/query/acc.cgi?acc=GSE131780 (accessed on 12 October 2025)”.

## Supplementary Materials

The following supporting information can be downloaded at: https://www.mdpi.com/article/10.3390/genes16121398/s1, Figure S1. Cell Cycle Scoring A: Distribution of cell cycle scores for LECs in the Sham group; Figure S2. Communication status of VEGF signal between clusters; Figure S3. LEC cell-cell interaction analysis; Figure S4. LEC and T cell communication analysis.All data supporting the findings of this study are available within the paper and its Supplementary Information. The following source data have been provided as supplementary files: Processed single-cell RNA-seq count matrices; Complete tables of differential expression statistics; Results of the cell–cell communication analysis. The online version contains supplementary material available at: Mendeley Data, Dataset Title: Supplement files for ‘Dysfunction and Pathological Origins of Lymphatic Endothelial Cells in Atherosclerosis Revealed by Single-Cell Transcriptomics’ “https://data.mendeley.com/datasets/dbszz6wbdy/1 (accessed on 13 November 2025)”.

## Abbreviations

The following abbreviations are used in this manuscript:

LEC	Lymphatic endothelial cell
VEC	Vascular endothelial cell
FB	Fibroblast
AS	Atherosclerosis
