# Supplementary material for: Dysfunction and Pathological Origins of Lymphatic Endothelial Cells in Atherosclerosis Revealed by Single-Cell Transcriptomics
Source: Genes (Basel). 2025 Nov 21;16(12):1398. doi: 10.3390/genes16121398 (PMC12733261; doi:10.3390/genes16121398)
Supplement: Supplementary file 1 [file genes-16-01398-s001.zip › supplement figures.pdf]

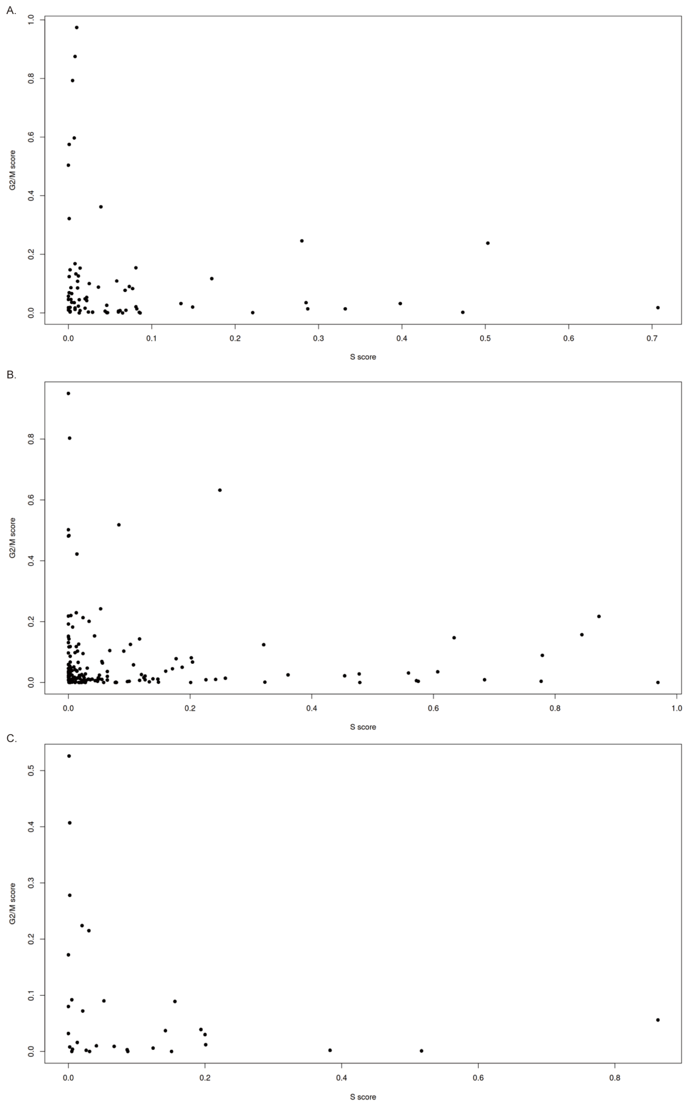

Figure S1. Cell Cycle Scoring A: Distribution of cell cycle scores for LECs in the Sham group. The x-axis and y-axis represent the S phase and G2/M phase scores, respectively. Cells with an S phase score > 0.4 and a G2/M phase score < 0.4 were classified as S phase cells. Cells with an S phase score < 0.4 and a G2/M phase score > 0.4 were classified as G2/M phase cells. Cells with both S phase and G2/M phase scores < 0.4 were classified as G1/G0 phase cells. B: Distribution of cell cycle scores for LECs in the AS1:8w group. C: Distribution of cell cycle scores for LECs in the AS2:16w group.

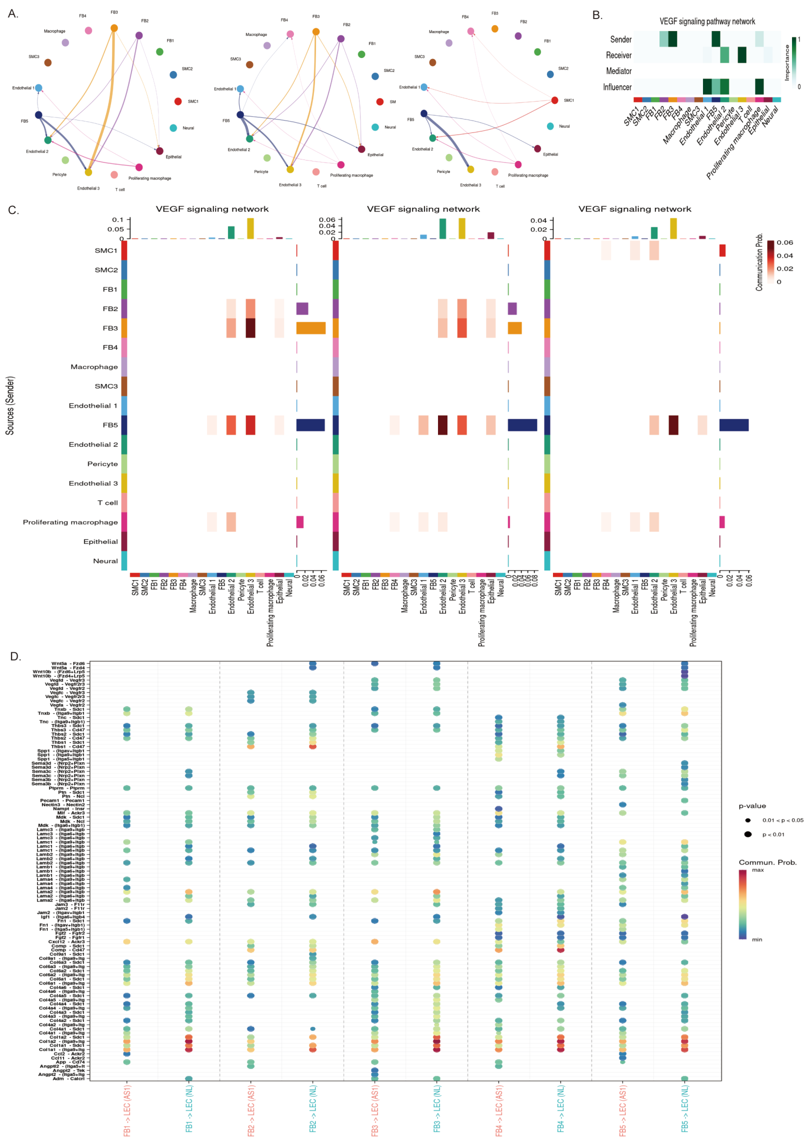

Figure S2. Communication status of VEGF signal between clusters. A: Overall cell-cell communication network (undirected, not distinguishing incoming/outgoing signals). Endothelial 3 represents the lymphatic endothelial cell (LEC) population; Endothelial 1 & 2 represent vascular endothelial cell (VEC) populations. Panels from left to right correspond to the Sham, AS1:8w, and AS2:16w groups, respectively. B-C: VEGF signaling network analysis. Sender represents the source cell populations secreting the signaling factors; Receiver represents the cell populations expressing the corresponding receptors. C: The y-axis represents the identity of the signal Sender, and the x-axis represents the identity of the Receiver. Panels from left to right correspond to the Sham, AS1:8w, and AS2:16w groups. D: Analysis of interactions between fibroblasts (FBs) and lymphatic endothelial cells (LECs), with FBs as the Sender and LECs as the Receiver. The top 100 receptor-ligand pairs by interaction strength are shown.

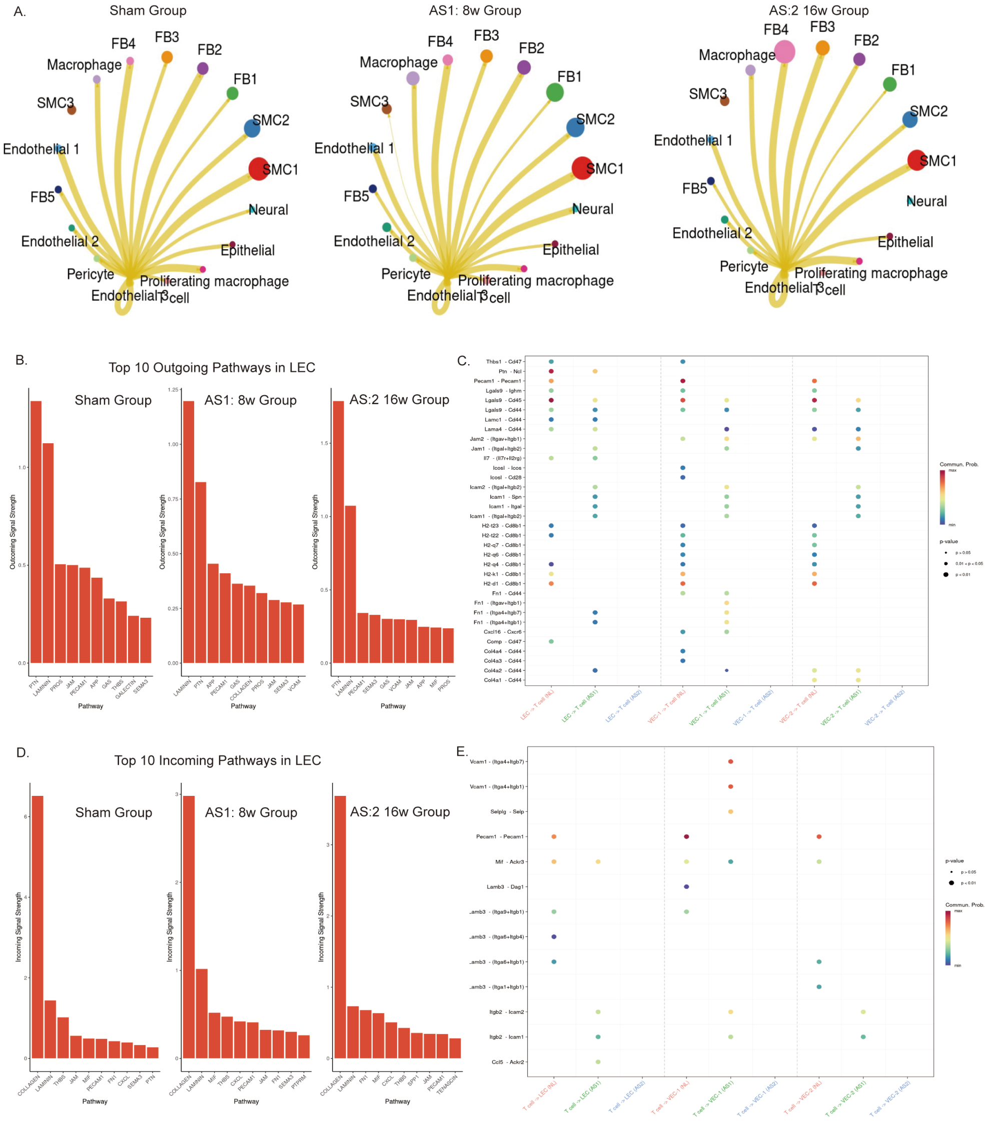

Figure S3. LEC cell-cell interaction analysis. A: Analysis of outgoing interactions from lymphatic endothelial cells (LECs, indicated as Endothelial3 in the figure). B: Top 10 outgoing signaling pathways from LECs. C: Analysis of interactions from LECs to T cells (NL: Sham group; AS1: AS1-8w group). D: Top 10 incoming signaling pathways to LECs. E: Analysis of interactions from T cells to LECs.

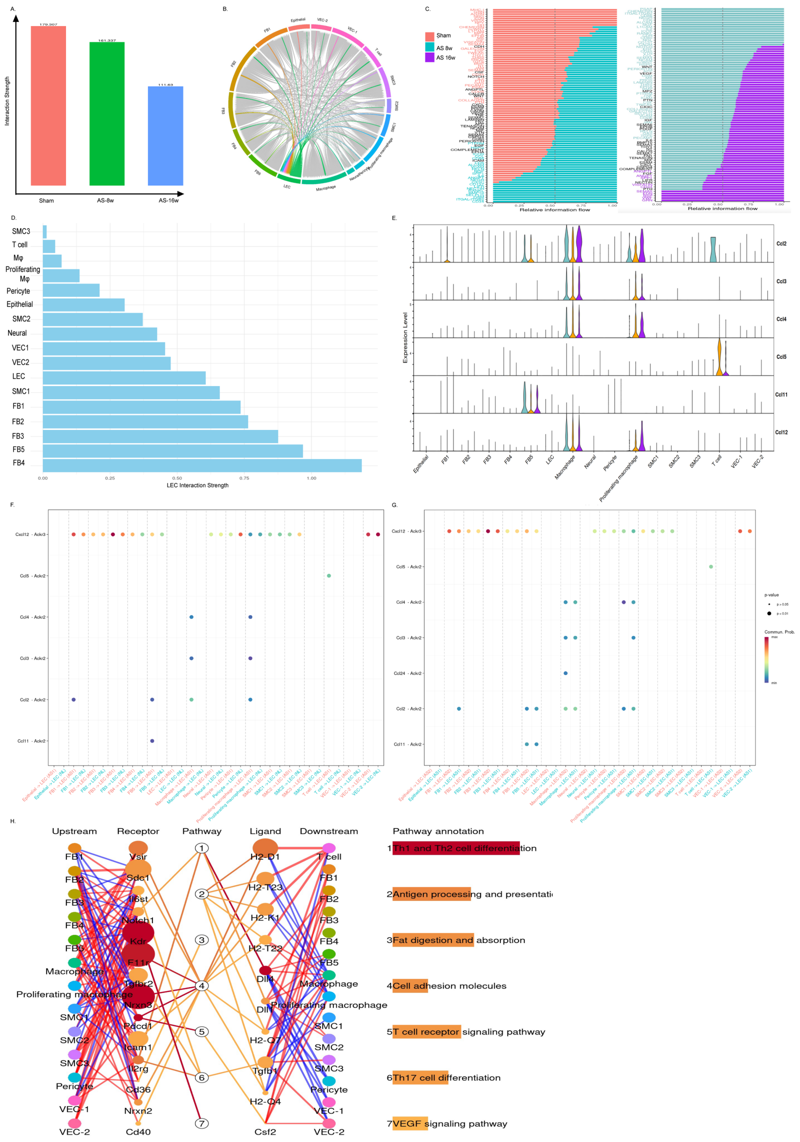

Figure S4. LEC and T cell communication analysis. A: Total cell-cell communication strength. B: Overall communication analysis for LECs (distinguishing incoming and outgoing signals). C: Differential cell communication analysis between groups. Red: Sham group; Blue: AS1-8w group; Purple: AS2-16w group. Highlighted pathways correspond to those with higher communication strength in the respective group. D: Ranking of cell subpopulations interacting with LECs, ordered from lowest (top) to highest (bottom) interaction strength. E: Violin plots showing expression changes of selected inflammatory chemokines. The primary secretory population for these chemokines is macrophages. F-G: Analysis of chemokine signaling regulation in LECs. NL: Sham group; S1: AS1-8w group; S2: AS2-16w group. H: Analysis of upstream and downstream communication partners for LECs. Red indicates higher communication strength in the Sham group; blue indicates higher communication strength in the AS1:8w group.
